# P-1571. Variability in Case-Ascertainment Methodology and Rates of Adult Invasive Pneumococcal Disease using a US Healthcare Claims Database

**DOI:** 10.1093/ofid/ofaf695.1751

**Published:** 2026-01-11

**Authors:** Ahuva Averin, Mark Rozenbaum, Stephen I Pelton, Rotem Lapidot, Amanda C Miles, Lindsay Grant, Alexander Lonshteyn, Maria J Tort, Jeffrey T Vietri, Derek Weycker

**Affiliations:** Avalere Health, Boston, Massachusetts; Pfizer Inc., Randstad, Noord-Holland, Netherlands; Boston Medical Center, Boston, Massachusetts; Boston Medical Center, Boston, Massachusetts; Pfizer, New York, NY; Pfizer Inc., Randstad, Noord-Holland, Netherlands; Avalere Health, Boston, Massachusetts; Pfizer, Inc, Collegeville, Pennsylvania; Pfizer, Inc., Collegeville, Pennsylvania; Avalere Health, Boston, Massachusetts

## Abstract

**Background:**

Healthcare claims databases are commonly employed to evaluate the epidemiology, outcomes, and costs of disease. However, validated case-ascertainment methods are largely unavailable, and thus outcome definitions often differ in terms of codes, algorithms, and/or data fields used to identify patients with a given disease; consequently, findings across studies often vary. In this study, we explored—as an exemplar—the impact of alternative case-ascertainment methods on variability in rates of adult invasive pneumococcal disease (IPD).

**Figure:**
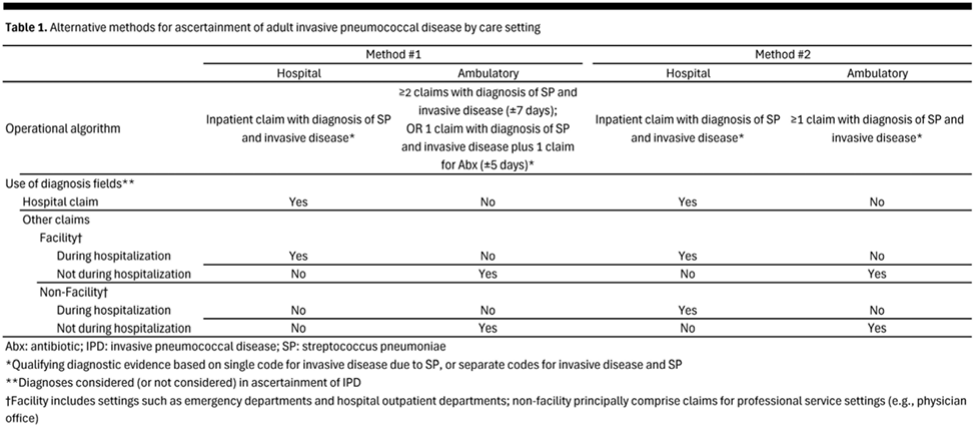

**Figure:**
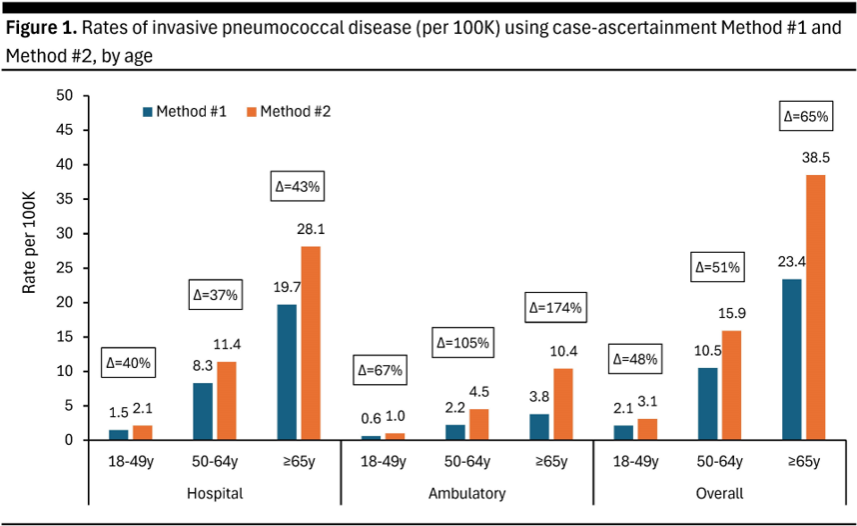

**Methods:**

A retrospective cohort design and the Optum Clinformatics DataMart (CDM; 2016-2019) were employed; the Optum CDM includes healthcare claims from a large commercial health plan in the United States. From the study population comprising adults aged ≥ 18 years, patients with IPD requiring hospitalization or ambulatory care only were identified using several alternative methods, which varied based on the algorithms and data fields (Table 1). Rates were calculated within age-specific subgroups, on an overall basis and by care setting, and were expressed per 100,000 person years (PYs). Results for two of the algorithms are presented herein.

**Results:**

With Method #1, which limited attention to diagnoses from hospital/facility claims during the admission, rates of IPD requiring hospitalization ranged from 1.5 to 28.1 per 100,000 PYs across age groups; with Method #2, which also included diagnoses from non-facility claims during the admission, hospitalized IPD rates were higher by 37-43% (Figure 1). For IPD requiring ambulatory care only, rates were higher by 67-174% when case-ascertainment was based on a single ambulatory claim with diagnostic evidence, versus ≥ 2 claims with diagnostic evidence and ≥ 1 claim for antibiotic therapy.

**Conclusion:**

Using adult IPD as an exemplar, findings from this evaluation suggest that practical differences in case-ascertainment methods may lead to large variability in disease rates from analyses utilizing healthcare claims databases. Findings also reinforce the need for validation studies and use of standardized methods.

**Disclosures:**

Ahuva Averin, MPP, Pfizer Inc: Grant/Research Support Mark Rozenbaum, PhD, M.B.A., Pfizer: Stocks/Bonds (Public Company) Stephen I. Pelton, MD, CSL Seqirus: Advisor/Consultant|GSK: Grant/Research Support|GSK: Honoraria|Merck Vaccines: Grant/Research Support|Merck Vaccines: Honoraria|Pfizer, Inc.: Grant/Research Support|Pfizer, Inc.: Honoraria|Sanofi: Honoraria|Sanofi: DSMB, Adjudicator for RSV vaccine trial Rotem Lapidot, MD, MSCI, Merck: Advisor/Consultant|Merck: Grant/Research Support|Merck: Honoraria|Pfizer: Advisor/Consultant|Pfizer: Grant/Research Support|Pfizer: Honoraria Amanda C. Miles, MPH, Pfizer: Employee of Pfizer Inc.|Pfizer: Stocks/Bonds (Public Company) Lindsay Grant, PhD, MPH, Pfizer: Employee|Pfizer: Stocks/Bonds (Private Company) Alexander Lonshteyn, PhD, Pfizer: Grant/Research Support Maria J. Tort, PhD, Pfizer, Inc: Stocks/Bonds (Public Company) Jeffrey T. Vietri, PhD, Pfizer Inc: Employment|Pfizer Inc: Stocks/Bonds (Public Company) Derek Weycker, Ph.D., Pfizer Inc.: Grant/Research Support

